# Gastric Epithelial Polyps: Current Diagnosis, Management, and Endoscopic Frontiers

**DOI:** 10.3390/cancers16223771

**Published:** 2024-11-08

**Authors:** Diego Reyes-Placencia, Elisa Cantú-Germano, Gonzalo Latorre, Alberto Espino, Glòria Fernández-Esparrach, Leticia Moreira

**Affiliations:** 1 Department of Gastroenterology, Faculty of Medicine, Pontificia Universidad Católica de Chile, Santiago 8320165, Chile; 2 Department of Gastroenterology, Fundació de Recerca Clínic Barcelona-Institut d’Investigacions Biomèdiques August Pi i Sunyer (FRCB-IDIBAPS), CIBEREHD, 08036 Barcelona, Spain; 3 Facultat de Medicina, Universitat de Barcelona, 08036 Barcelona, Spain

**Keywords:** gastric polyp, cancer risk, endoscopy, *Helicobacter pylori*

## Abstract

Gastric polyps (GPs) are common luminal lesions of the gastrointestinal tract, frequently detected incidentally in 1–6% of all upper gastrointestinal endoscopy. With the increasing use of endoscopy, the diagnosis of GPs has risen, highlighting the need for a thorough understanding of their pathophysiology and associated cancer risks. This review focuses on epithelial GPs, particularly fundic gland polyps (FGPs), hyperplastic polyps (GHPs) and gastric adenomas (GAs), discussing their classification, epidemiology, histopathology, endoscopic diagnosis, and current management. FGPs are predominantly associated with proton pump inhibitor (PPI) use and are common in regions with low *Helicobacter pylori* (*Hp*) prevalence, whereas GHPs are linked with *Hp* infection and chronic gastritis. GAs, although less common, have a significant risk of malignant transformation, especially in larger lesions. Management strategies vary according to polyp type, size, and dysplastic features, with resection recommended for high-risk lesions. Advances in endoscopic techniques, particularly narrow-band imaging (NBI), offer promising tools for differentiating between polyp types, potentially reducing the need for routine biopsies in the low-risk GPs. This review underscores the role of advanced imaging endoscopic techniques for the accurate classification and individualized management of GPs to mitigate the risk of gastric cancer.

## Simple Summary

Gastric polyps (GPs) are frequent gastric lesions, usually diagnosed incidentally. As upper gastrointestinal endoscopy becomes more frequent, the prevalence of GPs increases, with implications in clinical practice, as some of them can present different grades of malignant risk. The more common polyps are the epithelial ones, with a predominance of fundic gland polyps (FGPs) and gastric hyperplastic polyps (GHPs), in which prevalence varies according to local epidemiology. Up to now, the general recommendation is that all GPs should have histological evaluation by biopsy. However, novel advances in endoscopic techniques could allow for a more expectant management based on histopathological prediction by optical diagnosis. Further research and refinement of these techniques are necessary before their application in the clinical management of GPs.

## 1. Introduction

Polyps are defined as luminal lesions that project into the mucosal surface of the gastrointestinal tract and are characterized according to their morphological and histological features [1,2]. Gastric polyps (GPs) represent an important cause of outpatient consultation and most of them are diagnosed incidentally, with an increment in their diagnosis in recent years due to the increased use of upper gastrointestinal endoscopy, as they are found in 1–6% of all gastroscopies [3,4,5]. The symptomatic cases are generally related to the polyp size (hemorrhage and anemia caused by erosion of the GP surface) and location (abdominal pain and gastric outlet obstruction, especially in prepyloric polyps) [6].

Based on their histological characteristics, GPs are divided into epithelial (fundic gland polyps, hyperplastic polyps, gastric adenomas, and hamartomas) and non-mucosal intramural polyps (mainly stromal tumors, leimyomas, inflammatory fibroid polyps, fibromas, lipomas, ectopic pancreas, and neurogenic, vascular, and neuroendocrine tumors) [7]. In this review, we will focus on the current diagnosis and management of epithelial GPs, with a special mention about the endoscopic features of GPs and the future perspectives of GP evaluation by upper digestive endoscopy.

Epithelial polyps account for 60–90% of GPs and are divided into epithelial surface-derived polyps and oxyntic gland-derived polyps [8], of which the most frequent are fundic gland polyps (FGPs) and hyperplastic polyps (GHPs) [9]. In developed countries, the predominant type is FGPs, mainly associated with the use of proton pump inhibitors (PPIs), and in regions with a higher prevalence of *Hp* infection, the predominant types are GHP or adenomatous/neoplastic polyps [8].

It is known that the *Hp* prevalence can influence the kind of GP found among different regions. Currently, the worldwide prevalence of this infection is around 44%, with a variable distribution that can be higher than 80%, as observed in countries such as Colombia (83.1%), Nicaragua (83.3%), Ecuador (85.7%), Guatemala (86.6%), and Jordan (88.6%). Also, there are countries with a very low prevalence of *Hp*, as found in Hong Kong (15%), Indonesia 14.4%), Croatia (13.3%), New Zealand (9.2%), and Finland (9.1%) [10]. Unfortunately, there are no studies directed specifically to evaluate the prevalence of GPs among different *Hp*-prevalent regions.

It is important to know the different types of GPs, as the risk of progression to gastric cancer is variable among them, in order to establish appropriate treatment and surveillance [9,11]. The main characteristics of GPs are presented in Table 1 [6,8,12,13,14,15,16].

## 2. Fundic Gland Polyps

Also known as Elster’s glandular cysts, FGPs are the most frequent polyps (13–77%) in Western countries with a low prevalence of *Hp* infection [11,17,18]. Most of them are sporadic, with a clinical presentation of multiple polyps (but fewer than 10) more frequent in women and generally diagnosed at 40–60 years of age [8] (Table 1).

In a minority, FGPs are associated with polyposis syndromes, such as familial adenomatous polyposis (FAP), *MUTYH*-associated polyposis (MAP) and gastric proximal adenocarcinoma and polyposis syndrome (GAPPS). In the syndromic context, the polyps are numerous, sometimes with a “carpet” distribution, with an earlier presentation (between 20 and 40 years) and without sex predominance [8].

### 2.1. Pathophysiology and Histopathology

The sporadic FGPs are usually caused by activation of the somatic mutation of the beta catenin gene and arise because of exposure to certain risk factors, such as proton pump inhibitor (PPI) use [12,19]. The mechanisms are not exactly known but could be related with cystic dilations consequently to stagnation of fluid in the oxyntic glands. On the other hand, FGPs due to PPIs had a higher percentage of proliferating cells evaluated by Ki67 in comparison to sporadic FGPs [3]. The presence of FGPs in association with PPIs tends to increase as the time of exposure to the medication progresses, as evidenced in a meta-analysis of 12 studies, with an odds ratio (OR) of 4.71 (95% CI 2.22–9.99) for more than 6 months of PPI use and an OR of 5.32 (95% CI 2.58–10.99) for 12 months of PPI use [20]. Retrospective studies have reported a mean time of PPI use of 32.5 months for the development of FGPs and polyps tend to disappear within the first three months of PPI discontinuation. In addition, the occurrence of FGPs correlates with female sex (OR 1.77, 95% CI 1.31–2.39) and a lower risk of atrophic gastritis (OR 0.12, 95% CI 0.08–0.18) [21].

In addition, it has been proposed that competitive potassium acid blockers (P-CABs), such as Vonoprazan, could induce the hyperplasia of epithelial glands by mechanisms other than hypergastrinemia, with different histological features from PPIs [15].

The prevalence of *Hp* infection in patients with FGPs is usually very low due to a possible inhibitory effect on polyp development related to the presence of the bacterium [21,22].

**Figure 1:**
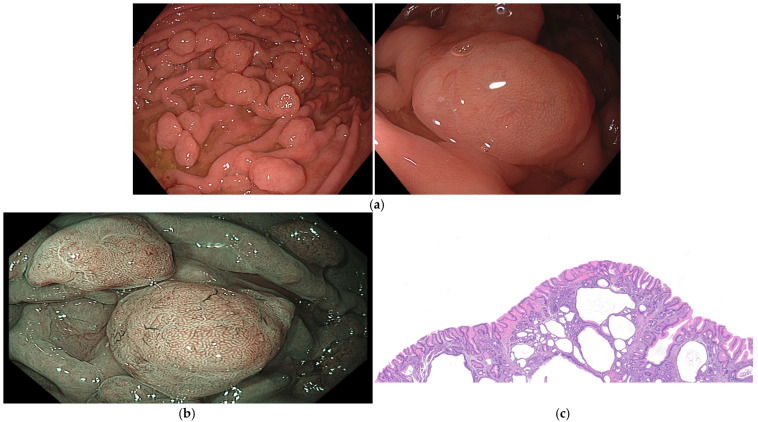
Fundic gland polyps (FGPs). (**a**) FGPs appear under white light endoscopy as small, smooth, sessile, pale, or translucent dome-shaped elevations, typically less than 5 mm in diameter; (**b**) FGPs evaluated with enhanced endoscopic image with narrow-band imaging (NBI) exhibit a distinctive whitish color against the darker background, featuring a characteristic “starfish” or “snowflake” surface pattern; (**c**) histopathologic features of FGPs, cystically dilated fundic glands aligned with parietal cells. Images were provided by Dr. Glòria Fernández-Esparrach (**a**,**b**) and Dr. Iván Arcilla (**c**).

The sporadic FGPs are mainly derived from oxyntic glands and are characterized by cystically dilated fundic glands aligned with parietal cells, chief cells, or foveolar/mucosal neck cells (Figure 1) [20].

In cases related to FAP, pathogenic germline variants in the *APC* gene have been observed in approximately 50% of cases. In GAPPS, pathogenic variants have been identified in the 1B promoter of the *APC* gene [8] and in the case of MAP, it is associated with pathogenic variants in the *MUTYH* gene [23]. These polyps have a smaller size and present microcysts, aligned with the fundic epithelium, without evidence of parietal cell or foveolar hyperplasia [8].

Dysplastic transformation is rare in non-syndromic sporadic cases, with an incidence of 1%, with just a few cases of progression to adenocarcinoma reported in the current literature [3,24]. On the other hand, in the syndromic cases, the rate of dysplastic transformation is higher, especially in GAPPS [8,22].

Other characteristics of FGP-associated syndromes are briefly explained in Table 2 [22,23,25,26].

### 2.2. Endoscopic Features

FGPs are usually smaller than 10 mm, sessile (Paris 0-Is) or semi-sessile, hyperemic or with a similar color to the normal mucosa, translucent with a smooth and shining surface, and have small blood vessels visible on the surface and a round dotted surface pattern. The mucosa surrounding the polyp usually is normal, and they are commonly located in the gastric fundus and body [2,14,27].

**Figure 2:**
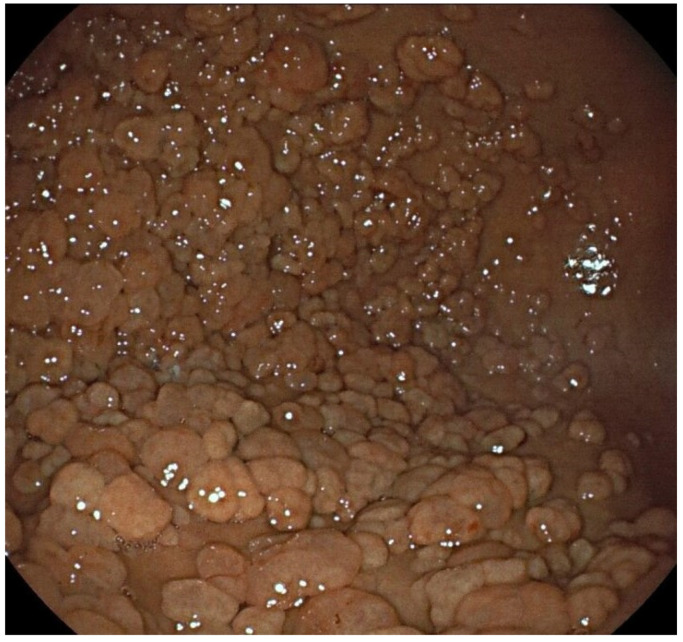
White light endoscopic (WLE) image: numerous fundic gland polyps in a “carpet” distribution in a patient with familial adenomatous polyposis. Images were provided by Dr. María Daca.

It is estimated that up to 25% of sporadic cases may present multiple polyps. In cases associated with hereditary syndromes, between 40 and 80% of patients with FAP and the majority of patients with GAPPS can present more than 100 polyps (Figure 2) [8]. The simultaneous presence of duodenal adenomas should alert to the possibility of FAP or MAP [25,26].

### 2.3. Management

Resection of these lesions is recommended in the presence of atypical features, such as polyps larger than 10 mm, antral location, ulceration, a surface with irregularity, depressed areas, erosions, or irregular vessels. In situations where resection is not feasible, directed biopsies can be considered. In patients with PPI use, the drug indication and dosage should be reviewed. Endoscopic follow-up is not necessary in non-syndromic sporadic cases [7,11].

However, a suspicion of a syndromic case should be made in patients under 40 years old, with more than 20 polyps and suspicion of dysplastic changes (atypical surface or vascular pattern). In these cases, a detailed evaluation of the duodenum and a colonoscopy should be performed to evaluate the possibility of polyposis syndrome. In case of a dysplastic FGP, it should be resected endoscopically and followed-up with an annual endoscopy [8,11,23].

## 3. Hyperplastic Polyps

GHPs are the second most frequent (17–55%) types of GP in the West, usually seen in patients during the sixth and seventh decade of life. They are commonly associated with underlying gastritis within the context of intestinal metaplasia (37%), *Hp* gastritis (25%), reactive or chemical gastropathy (21%), and autoimmune gastritis (12%). Other described associations are gastroesophageal reflux disease (GHPs can be found in the cardias) [3], Menetrier’s gastropathy [3,21], gastric antral vascular ectasia, and cytomegalovirus gastritis. Multiples polyps are found in 20% of cases; when there are more than 50 polyps, it is called hyperplastic polyposis [8,11,14].

### 3.1. Pathophysiology and Histopathology

Most studies describe the absence of somatic pathogenic mutations in small non-dysplastic GHPs, implying a true reparative/hyperplastic nature. It has been described that GHPs are present in up to 8.9% of chronic PPI users, especially with hypergastrinemia and concurrent *Hp* infection. It is believed that the influence of gastrin on the receptors expressed in the foveolar epithelium generates proliferative effects in the gastric mucosa, resulting in GHPs [20]. About the *Hp* infection, it is present in 6–20% of GHPs, and the polyps may disappear after *Hp* eradication. A recent meta-analysis showed that the cumulative clearance rate of GHPs after confirming *Hp* eradication was 79% (95% CI, 72–86%) [28]. Their mechanism was reported, showing that *Hp* promotes inflammation and reactive hyperplasia of the gastric mucosa with the formation of GHPs. The background of this phenomenon is higher serum gastrin levels, lower pepsinogen I, and higher pepsinogen II levels [29]. In this context, GHPs have been interpreted as a marker of increased risk for gastric cancer due to the relationship with *Hp* infection, gastric atrophy, and hypergastrinemia [3].

In larger polyps, genetic alterations have been identified, especially in those with dysplasia or adenocarcinoma. Abnormalities detected include loss of MGMT expression, somatic mutations in *APC* or *CTNNB1* (beta catenin), and less frequently, alterations in *KRAS* or *BRAF*. Some GHPs with dysplasia present mutations in TP53, and immunohistochemistry for this protein could help in differentiating reactive atypia versus dysplasia [8].

**Figure 3:**
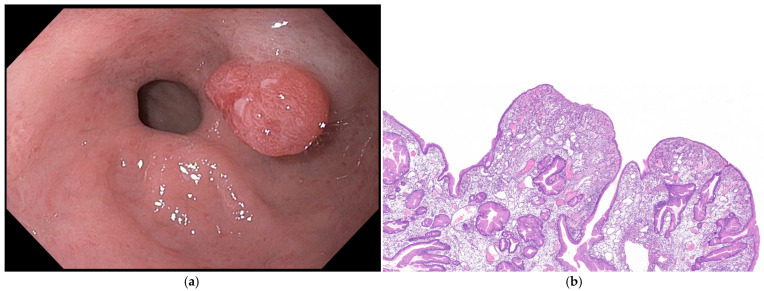
A hyperplastic polyp. (**a**) Macroscopic features with high-definition WLE: sessile polyp with a lobulated reddish surface and two erosions in the cup of the lesion; (**b**) histopathologic features of hyperplastic polyps with pyloric cells, chief cells, and parietal cells. Images were provided by Dr. Glòria Fernández-Esparrach (**a**) and Dr. Iván Arcilla (**b**).

Histologically, GHPs may have pyloric cells, chief cells, and parietal cells (Figure 3) [21]. They are associated with intestinal metaplasia in 4–16% and with dysplasia in 4%. The risk of neoplastic transformation in GHPs increases mainly in polyps larger than 20–25 mm, and the rate of focal carcinoma can vary between 0.8 and 10% [2]. Also, GHPs appear to be associated with a risk of up to 8.5% in the development of gastric cancer in the surrounding gastric mucosa [2].

### 3.2. Differential Diagnosis

Portal hypertension-associated gastric polyps (PHGPs) should be distinguished from GHPs and inflammatory fibroid polyps, as they are similar in appearance and histology. The first began due to vascular mucosal lesions and not by chronic inflammation as with GHPs; they usually occur between 43 and 70 years, can be single or multiple, and are located in antrum or gastric body, with size ranges from 2 to 30 mm. They tend to disappear after liver transplantation. There is no specific recommendation for PHGP management, but they can be treated with argon plasma coagulation or endoscopic resection if symptomatic [13].

Other polyps that should be distinguished from GHPs are FGPs, gastric adenomas, and gastric hamartomatous polyps with marked differences in their endoscopic mucosal pattern and in their histological features, as detailed in this review.

### 3.3. Endoscopic Features

GHPs are sessile (Paris 0-Is) or pedunculated (Paris 0-Ip), usually unique and located in the gastric antrum or adjacent to ulcers or lesions. They present a broad base and a smooth, lobulated outline, usually reddish. Also, they can present erosions or a whitish exudate (Figure 3) [21]. GHPs are generally lower than 20 mm, but can eventually grow bigger, and in cases larger than 25 mm, the risk of neoplastic transformation increases [8,30]. The mucosa surrounding the polyp usually presents inflammation [13].

### 3.4. Management

The diagnosis of GHPs should be confirmed by histological analysis. It is recommended to evaluate the stomach for synchronous neoplasia, define the degree and extent of atrophic gastritis, and diagnose *Hp*, which should be eradicated if detected [28].

GHPs larger than 5–10 mm, pedunculated or symptomatic (obstruction or bleeding/anemia), should be completely resected due to the increased risk of harboring malignancy [12,31]. Endoscopic follow-up will be determined depending on the polyp histology (presence or absence of dysplasia) and other risk factors for gastric cancer, such as family history, patients from areas with high gastric cancer risk, atrophic gastritis, and intestinal metaplasia (evaluated by updated Sydney protocol) [11,14,30].

## 4. Gastric Adenomas

Gastric adenomas (GAs) contribute to 1 to 10% of GPs. The most frequent age of presentation is in the seventh decade, without predominance by sex. They are frequently identified in the context of atrophic gastropathy and intestinal metaplasia (whether due to *Hp* or autoimmune gastritis), so they can be considered precursors of gastric adenocarcinoma. Also, the presence of GAs can be associated with synchronous and metachronous adenocarcinomas [3,11,30], with synchronous adenocarcinoma described in up to 30% of cases of GAs.

GAs present a risk of malignancy of 1.3%, higher than other epithelial polyps. Their malignant potential is influenced by the size of the polyp, especially cases larger than 20 mm, that can contain foci of adenocarcinoma in around 50%. Also, there is more malignant transformation in cases with high-grade dysplasia or with a villous component, with a risk that rises up to 50%. [21,30,32].

### 4.1. Pathophysiology and Histopathology

GAs can be divided into adenomas derived from epithelial surfaces (intestinal and foveolar) or derived from glandular tissue (pyloric or oxyntic) [2]. The intestinal and fundic gland types have a higher risk of progression to gastric cancer when compared to foveolar and oxyntic gland GAs [3].

a. - Adenomas derived from epithelial surface

Intestinal adenomas are positive for MUC2, CD10, and CDX2, while foveolar adenomas variably express MUC5AC and MUC6. Both accumulate alterations in *APC*, *KRAS*, *ERBB3*, and *TP3* and could present the inactivation of genes of the DNA repair system [8].

b. Adenomas derived from glandular tissue

In the case of pyloric gland adenomas, they develop in three scenarios, namely chronic atrophic gastritis (associated with autoimmune gastritis), hereditary predisposition syndrome (PAF and MAP), and sporadically in normal mucosa. Its immunohistochemical marker is the expression of MUC5. The most prevalent molecular alteration is GNAS (63 to 83%) and KRAS (41 to 67%), with possible overexpression of p53 and nuclear beta catenin [8].

The oxyntic gland adenoma expresses MUC6, with low Ki-67. In 50%, these lesions have mutations in the Wnt/beta catenin pathway, such as *CTNNB1*, *AXINs*, *APC*, or *GNAS* [8].

The association of GAs with hereditary syndromes such as PAF and MAP have been described, although much less frequently than with FGPs. The presence of these syndromes in association with *Hp* infection increases GA malignant risk, especially in the intestinal type [4,23].

In addition, dyslipidemia, alcohol consumption, and *Hp* infection independently increase the risk of developing adenomatous polyps [16].

### 4.2. Endoscopic Features

**Figure 4:**
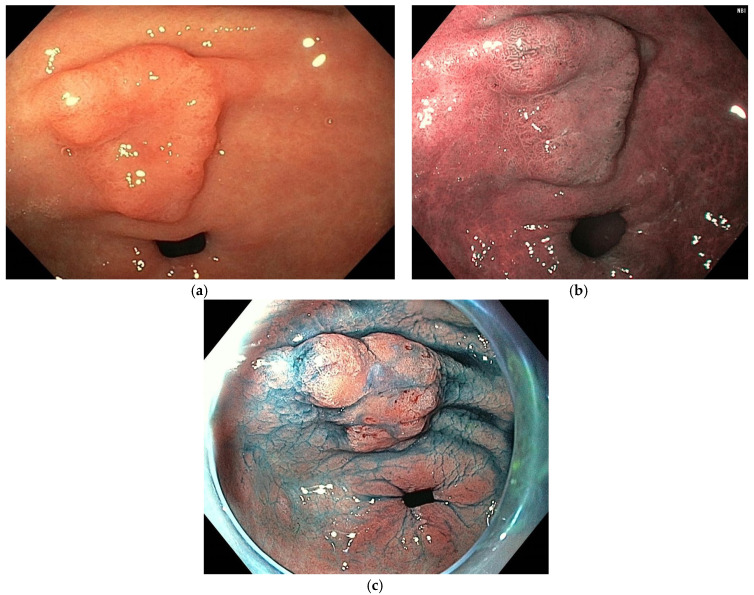
A gastric juxtapyloric adenoma. (**a**) A high-definition white light endoscopic image of a flat elevated lesion of 15 mm with a pink lobulated appearance and a central depression. (**b**) An endoscopic image with virtual chromoendoscopy (NBI), with some areas with a prolonged mucosal pattern; (**c**) an endoscopic image with conventional chromoendoscopy with indigo carmine. Images were provided by Dr. Glòria Fernández-Esparrach (**a**–**c**).

GAs are usually single (82%), smaller than 20 mm, and are located in the antrum and incisura. They present a pale lobulated appearance and can be sessile (Paris 0-Is), pedunculated (Paris 0-Ip), or elevated flat (Paris 0-IIa) lesions [21,30] (Figure 4). Surface patterns might be tubular or villous and an irregularity of the vascular pattern may suggest a higher grade of dysplasia. A commonly white opaque substance (WOS) can be observed in these lesions. It has been described that a regular arrangement of the WOS is associated with dysplasia, while an irregular arrangement is related to adenocarcinoma [33].

### 4.3. Management

In the suspicion of a GA, its diagnosis and degree of dysplasia should be confirmed by histologic analysis before performing treatment. An extensive evaluation of the stomach should be assessed because of the risk of synchronous neoplasia, atrophic gastritis, and intestinal metaplasia. *Hp* infection should be investigated and if identified, should be eradicated [3,14].

All GAs larger than 5 mm should be endoscopically resected, with complete excision, as this interferes in an adequate histological examination. The endoscopic submucosal dissection (ESD) is suggested for sessile adenomas larger than 15 mm, as these cases have more risk of invasive neoplasia; with ESD, *en bloc* excision is more feasible; consequently, the risk of recurrence is lower than with EMR [30].

Endoscopic follow-up should be performed 6–12 months after the GA resection to assess healing and detect any early recurrence. Patients should continue in gastroscopy surveillance at yearly intervals depending on the amount of GAs, their size, and the grade of dysplasia [4,12,14,21,30].

## 5. Gastric Hamartomatous Polyps

Hamartomatous polyps represent less than 1% of GPs. They are characterized by disorganized tissue growth and can be solitary or syndromic (such as Peutz Jeghers syndrome, juvenile polyposis, or Cowden syndrome) [21].

They are usually smaller than 10 mm and indistinguishable from hyperplastic polyps. When solitary, they are mostly located in the antrum, without malignant potential. In the case of multiple polyps, the usual involvement is in the gastric body, with malignant potential. The risk of gastric cancer occurs mainly in the context of Peutz Jeghers syndrome and in juvenile polyposis, since in Cowden syndrome, the gastric malignancy affects about 1% of patients [21].

Endoscopic removal should be performed in polyps larger than 5 mm, but in the syndromic context, clearing all of them may not be feasible. After the resection, an endoscopic follow-up should be performed at least once every 1–3 years, varying according to the associated syndrome [34].

## 6. Other Polyps

Even though gastric neuroendocrine tumors (NETs) are not of an epithelial origin, they are being identified in endoscopy with an increasing incidence (0.6–2% of GPs). Moreover, asymptomatic NETs are rising as a finding because of the use of endoscopy as part of screening for upper GI neoplasms. For those reasons, there will be a brief description about the principal characteristics of the NETs [21,35].

**Figure 5:**
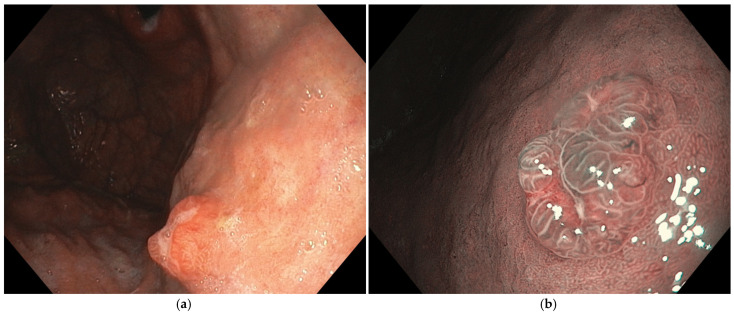
Gastric neuroendocrine tumors. (**a**) A high-definition white-light endoscopic image of a hyperemic sessile lesion of 15 mm with a central ulceration, surrounded by an atrophic appearance of the gastric mucosa. (**b**) Endoscopic image with virtual chromoendoscopy (narrow-band imaging) of the lesion. The vascular pattern has a centripetal distribution. Images were provided by Dr. Alberto Espino (**a**,**b**).

Gastric NETs have an annual incidence of 0.4/100,000 patients, with a prevalence of 3/100,000. They are classified into the three following subtypes:

- Type I: They develop in the presence of chronic atrophic gastritis associated with hypergastrinemia (especially in the context of autoimmune gastritis). It accounts for 75 to 90% of all gastric NETs. They are usually asymptomatic and more common in women. They are characterized by being smaller than 10 mm, hypervascularized, and sessile; some present a central depression or erosion, multiplicity and located in the gastric fundus and body. Their histology consists of clusters of endocrine cells with low Ki67. In cases smaller than 10 mm, annual/biennial endoscopic follow-up is suggested, and those larger than 10 mm should be removed. If recurrent or with multiple or invasive lesions, antrectomy may be an alternative (Figure 5).

- Type II: Associated with hypergastrinemia derived from gastrin-secreting tumors (Zollinger Ellison syndrome) and associated with multiple endocrine neoplasia type 1. It has macroscopic features similar to type I, with normal-appearing surrounding mucosa, and it is the least frequent type (5–8% of gastric NETs). All type II NETs should be resected due to the risk of nodal involvement and metastasis, with resection of the associated gastrinoma and annual endoscopic surveillance.
- Type III: Without hypergastrinemia and sporadic. They are single, large, 20 to 50 mm, and they originate in healthy gastric mucosa and in any area of the stomach, representing 15 to 20% of gastric NETs. They have the worst prognosis and are usually symptomatic, with Ki67 over 20%, a high percentage of metastasis. They require surgical resection and adjuvant therapy similar to gastric adenocarcinoma.

In all cases, surgery is suggested in cases with lesions over 20 mm, suspicion of muscularis propria invasion, high Ki67, and lymphovascular infiltration [36,37].

## 7. Advances in Endoscopic Evaluation of Gastric Polyps

There are some limitations about an adequate histological diagnosis of a GP and the use of forceps biopsy. In a prospective multicenter study of Muehldorfer et al., endoscopically removable GPs (>5 mm) underwent forceps biopsy and were completely resected by polypectomy; then, the histological results of both procedures were compared. A correct histological diagnosis could be obtained without a complete polypectomy in 97.3% of the cases but foci of the carcinoma in hyperplastic polyps may be missed with biopsy sampling. Previous experiences reported a discrepancy rate between biopsy and polypectomy results that ranged from 0 to 29% [38]. Those reasons reinforce the importance of a morphological description to perform proper management when a GP is found. First, polyp morphology should be classified according to the Paris classification [30,39].

Narrow-band imaging (NBI) is an electronic chromoendoscopy based on a technique that uses a spectral narrow-band filter, which enhances the visualization of microvascular and mucosal patterns, resulting in an image with a bigger contrast between vessels and the surrounding mucosa [40,41]. In the last few years, some studies have shown evidence that NBI with magnification can be useful for predicting GP histopathology [31,41].

**Figure 6:**
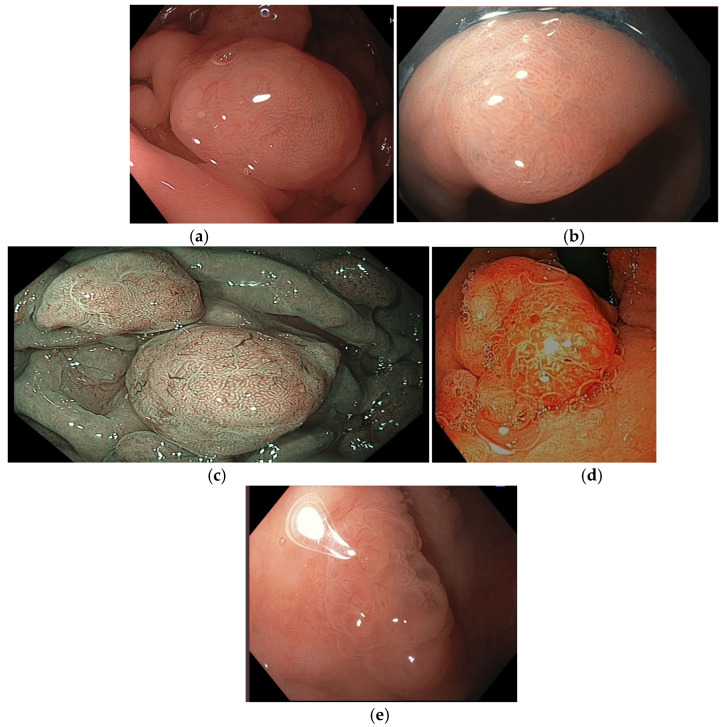
Mucosal and microvascular patterns. (**a**) Prolonged mucosal pattern; (**b**) villous mucosal pattern; (**c**) honeycomb microvascular pattern; (**d**) dense vascular pattern; and (**e**) core vascular pattern. Images were provided by Dr. Glòria Fernández-Esparrach (**a**,**c**,**d**) and Dr. Gonzalo Latorre (**b**,**e**).

Omori et al. classified gastric polypoid lesions by mucosal pattern into four categories (small round; prolonged; villous or ridge; unclear) and by microvascular patterns into five categories (honeycomb; dense vascular; fine network; core vascular; unclear) (Figure 6). In their analysis for predicting FGPs and GHPs, the most suggestive patterns were the combination of small round/honeycomb (sensitivity 94.7%; specificity 97.4%) and prolonged and villous or ridge/dense vascular patterns (sensitivity 93.6%; specificity 91.6%), respectively. Contrarily, the most favorable combined patterns for gastric neoplasia (GN) prediction were small round/fine network; prolonged/core vascular; villous or ridge/core vascular; unclear/core vascular; and unclear/unclear (sensitivity 86.2%; specificity 97.0%). Interestingly, 4/48 cases with a dense vascular pattern (suggestive of GHPs) contained a small component of well-differentiated adenocarcinoma, and all these cases had fine mucosal structures with villous or ridge patterns and were 5 mm or larger in size [31].

Takahashi et al. evidenced a vessel dilation exhibiting branching architecture both in GAs and in FGPs. They observed in magnifying endoscopy with NBI that the presence of a ring-shaped white zone surrounding the polyp, known as a white ring sign (WRS), could be used as a reliable indicator for FGPs. So, WRS positivity in a polyp with a regular microsurface and microvascular patterns could mean that further evaluation and treatment may not be necessary. At the same time, the absence of a WRS suggests the possibility of GAs, requiring additional evaluations, such as endoscopic ultrasonography and endoscopic resection [42].

However, the limitation of the previous classification systems is that they use magnification. In the pilot study of Esposito et al., gastric polypoid lesions were evaluated with NBI without magnification and categorized by five variables (mucosal and vascular pattern, vascular thickness and density, presence of light-blue crest), and they found that a regular tubule villous mucosal pattern was suspicious of adenoma, so biopsies should be performed. On the other hand, in case of a regular circular mucosa without any central erosion, the likelihood of being a GHP was high, so no biopsies were needed [40].

At the same time, in the USA, a simple descriptive classification was developed based on existing classification schemes and using nomenclature from the NBI International Colorectal Endoscopic classification as a guide. Asztalos et al. used three characteristics to describe the GP by NBI, which were color (same as/lighter than background or browner relative to background), vessel pattern (no vessels, isolated lacy vessels, or brown vessels), and surface pattern (homogenous absence of pattern; dark or white spots of uniform size; oval, tubular, or white structures surrounded by brown vessels). Then, they proposed an algorithm for differentiating low-risk polyps from high-risk polyps. They identified low-risk polyps more frequently in the polyps with a homogenous absence of surface patterns or isolated lacy vessels (100% negative predictive value—NPV). Also, the vessel pattern was the most effective at differentiating FGPs from other lesions; isolated lacy vessels cleared the 90% NPV threshold for differentiating FGPs. In conclusion, they suggest the future possibility of a “diagnose-and-leave” strategy for GPs smaller than 10 mm with an optical NBI diagnosis of FGPs or GHPs with low-risk features [43].

The combination of NBI and magnifying endoscopy has also shown to be useful for the characterization of GAs when a demarcation line, numerous whitish and slit-like crypt openings, or a white opaque substance (WOS) are found [33,44]. The presence of a WOS in a GHP may be considered an endoscopic finding that is predictive of the neoplastic transformation of the polyp [45].

Chromoendoscopy with acetic acid can also be used, as shown in the report of Lafeuille et al., where the loss of the acetic whitening indicated the presence of neoplasia in a GHP [46].

Nowadays, the application of electronic chromoendoscopy is becoming more available in clinical practice, but it is necessary to have a specific level of expertise to perform and to interpretate its images, and it is most beneficial when performed by trained professionals. To achieve optimal outcomes in GP differentiation by virtual or dye-based chromoendoscopy, comprehensive training programs conducted in a secure and standardized setting are essential to develop the requisite skills [47].

In terms of novel advances with artificial intelligence (AI) in digestive endoscopy, up to now, most studies are directed towards the evaluation of colorectal polyps, early esophageal cancer, and early gastric cancer [48,49,50,51]. Specifically, in terms of GPs, so far, there are only a few studies that are directed at general GP detection [48,52,53,54,55]. To the best of our knowledge, there are no reports describing an AI system that could assist endoscopists in differentiation among the types of GPs and to predict their malignancy.

The role of endosonography in gastric polyps remains unclear. However, in cases of potential subepithelial lesions, it proves highly useful, especially in challenging diagnoses, where it enables better characterization than conventional digestive endoscopy and assists in assessing the need for targeted biopsies [56].

## 8. Discussion

The present review has provided an overview of GPs, focusing on the subtypes, their clinical significance, endoscopic characteristics, and management strategies. The classification of GPs into different histological types, including FGPs, GHPs, and GAs, highlights the importance of understanding their pathophysiology and potential for malignant transformation. This review emphasizes the varying risks associated with each type and the importance of the differential diagnosis with other gastric lesions such as NETs.

One key point is the association between certain GPs and specific risk factors or syndromes. For instance, FGPs are often linked to PPI use and may exhibit different characteristics when associated with polyposis syndromes such as FAP or GAPPS. This raises important questions, as some genetic factors contribute to the development of GPs and whether certain subpopulations may benefit from different management strategies.

Another crucial aspect is the pathophysiological distinction between sporadic and syndromic cases. The low rate of dysplastic transformation in sporadic FGPs contrasts with higher risks observed in some syndromes, particularly with GAPPS. This difference highlights the value of identifying syndromic presentations early, as it may prompt more aggressive surveillance.

Regarding GHPs, their association with chronic gastritis, *Hp* infection, and hypergastrinemia is important in their management. Their presence in patients with *Hp* suggests that eradication therapy could be a preventative measure against polyp recurrence while also serving as a marker of increased gastric cancer risk. Further research is needed to delineate molecular pathways involved in the progression of larger GHPs to dysplasia.

The review also emphasizes the role of endoscopic techniques, particularly the use of electronic chromoendoscopy, to enhance the analysis of vascular and mucosal patterns. While the traditional endoscopic biopsy approach has limitations in accurately predicting the histopathology of GPs, the evolving use of electronic chromoendoscopy may provide a non-invasive test to differentiate among GP subtypes. However, further validation of classification systems for endoscopic appearance, as well as standardized training for endoscopists, is necessary to improve diagnostic accuracy.

**Figure 7:**
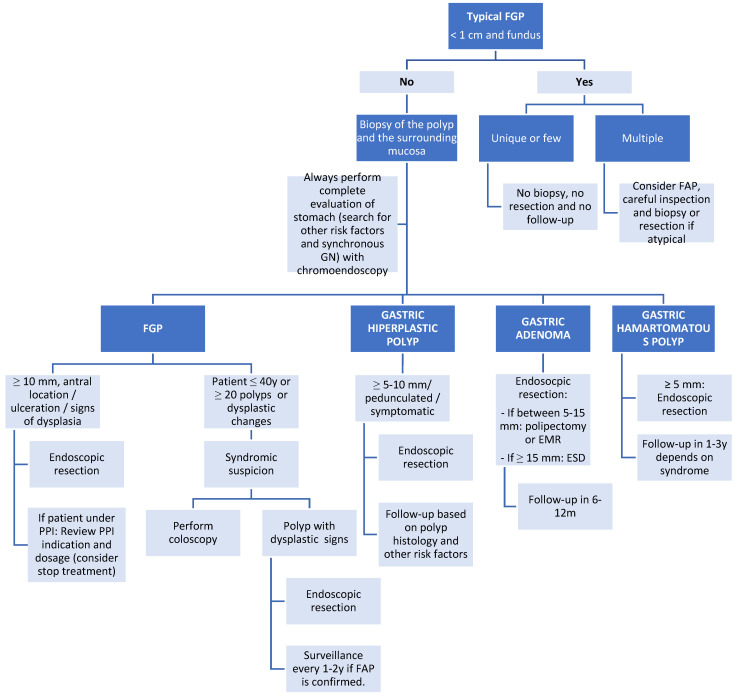
Management of gastric polyps.

Overall, this review highlights the complexity of managing GPs due to the heterogeneity in their types, underlying etiologies, and malignant potential. Continued research and consensus on the classification and treatment of GPs are essential for optimizing patient outcomes and preventing gastric cancer development. An algorithm of the current management of GPs is proposed according to previous information (Figure 7) [21,30].

## 9. Conclusions

GPs represent a diverse group of gastrointestinal lesions with varying risk of harboring malignancy and potential for malignant transformation over time, so a nuanced approach to their diagnosis and management is necessary. This review highlights the importance of distinguishing between the different types of epithelial polyps, such as FGPs, GHPs, and GAs, each of which presents unique pathological features and malignant risk.

## 10. Future Directions

Nowadays, advances in chromoendoscopic technology have been demonstrating the ability to predict GP histology and identify high-risk lesions, potentially reducing the need for invasive procedures. Effective management, including appropriate surveillance and endoscopic resection of high-risk polyps, is crucial in preventing the progression to gastric cancer.

These new studies about the endoscopic NBI characterization of GPs show that this technique can be a useful tool in the real-time endoscopic differentiation of benign to pathologic GPs, as we currently perform in colonic polyps. Regarding the application of AI in digestive endoscopy, specifically in GP differentiation, studies are in a very early phase. This way, further research and the refinement of NBI and AI devices are necessary to fully realize their potential in clinical practice to prioritize the therapeutic management of GPs with high suspicion of advanced histology and avoid biopsies in GPs without any suspicion of advanced histology. As endoscopic techniques continue to evolve, they hold promise for a more precise and less invasive management of GPs, preventing unnecessary costs in medical practice and most of all, improving patient outcomes.

Moreover, there could be non-invasive markers for the detection of circulating tumor cells in gastric cancer patients and often upregulated in early stages, such as some miRNAs and the use of the traditional markers (CEA and CA 19-9). Those laboratory tests could be used in the presence of GPs to predict their cancer risk [57]. In addition, miRNA-1290 plays a role in numerous signal regulation mechanisms, with an increase in its expression during *H. pylori*-induced gastric lesions. CagA induces an increase in miRNA-1290 levels, which affects the expression of tumor suppressor genes and results in malignant lesions, with a possible role in polyps associated with *H. pylori* infection [58]. Further investigations are needed.

## Figures and Tables

**Table 1:** Main characteristics of gastric polyps.

Features	Fundic Gland Polyps	Hyperplastic Polyps	Gastric Adenomas	Hamartomas
**Frequency**	47–77%.	17–55%.	1–10%.	Less than 1%.
**Age/sex distribution**	Sporadic: middle aged/women. Syndromic: early age/no sex difference.	Middle aged. No sex difference.	Middle aged. Men (in the case of intestinal type).	Children and young age.
**Endoscopic features**	Often multiple (but fewer than 10 in sporadic cases). Numerous polyps if syndromic. Smaller than 10 mm. Sessile, hyperemic, translucent, and smooth surface.	Usually single or few. Lower than 20 mm. Sessile or pedunculated. Smooth or lobulated, reddish appearance with whitish exudate.	Single (82% of cases). Smaller than 20 mm. Sessile or pedunculated, pale color.	Single (sporadic) or multiple (syndromic). Smaller than 10 mm. Indistinguishable from hyperplastic polyps.
**Location**	Fundus and gastric body.	Antrum. Adjacent to ulcers or lesions.	Antrum and incisura angularis.	Antrum (solitary) and gastric body (multiple polyps).
**Associated conditions**	Chronic use of PPIs. Hereditary syndromes (PAF, MAP, GAPPS).	*Hp* infection. Autoimmune gastritis. Chronic atrophic gastritis.	Hereditary syndromes (PAF, MAP). Dyslipidemia. Alcohol consumption. *Hp* infection.	Hereditary syndromes (Peutz Jeghers syndrome, juvenile polyposis, Cowden syndrome).
**Histopathology**	Large fundic gland cysts with parietal, chief, and some mucous cells.	Foveolar epithelium with apical neutral mucin layer. Foveolar cells may develop hypertrophic features, with clusters of pseudocaliciform cells or signet ring cells. Lamina propria may have lymphoplasmacytic and eosinophilic infiltration with edema.	Intestinal adenoma: pseudostratified columnar epithelium with variable number of goblet cells. Foveolar adenoma: columnar or cuboidal foveolar epithelium with apical neutral mucin layer. Pyloric gland adenoma: narrow uniform tubules with low columnar to cuboidal monocellular epithelium. Oxyntic gland adenoma: predominant chief cell pattern, with parietal cells and mucous neck cells. Mild-to-moderate nuclear atypia may be present.	Disorganized tissue growth. Peutz Jeghers syndrome: nondysplastic overlying gastric epithelium, with arborizing pattern of growth with muscularis mucosae extending into branching fronds of the polyp. Juvenile polyposis: abundant lamina propria with benign but often elongated and cystically dilated glands and lack of smooth muscle core.

**Table 2:** FGP-associated syndromes.

Syndrome	Familial Adenomatous Polyposis	*MUTYH*-Associated Polyposis	Gastric Adenocarcinoma and Gastric Proximal Polyposis
**Inheritance**	Autosomal dominant disorder.	Autosomal recessive disorder.	Autosomal dominant disorder with incomplete penetrance.
**Genetic mutation**	Germline pathogenic variant in the *APC* gene. Up to a quarter of patients may have a de novo mutation.	Germline biallelic pathogenic variant in the *MUTYH* gene.	Three-point mutations in the B1 promoter of the *APC* gene.
**Incidence**	Incidence of 1/8300 births.	1–2% general population.	Incidence and prevalence still unknown.
**Gastrointestinal phenotype**	Stomach: FGPs found in 65–88%. GAs found in 14%. Frequent duodenal polyps. Hundreds of thousands of colorectal adenomas.	Gastric polyps are less common than in FAP (found in 11%). - FGP—52.4% - GA—24%. Simultaneous duodenal adenomas may occur. Increased risk of colorectal cancer.	Fundic gland polyposis restricted to body and fundus. Significant risk of intestinal-type gastric adenocarcinoma. Without duodenal or colonic polyposis. May have a higher risk of sporadic colonic adenomas than general population.
**Specific surveillance and treatment**	Gastroduodenoscopy surveillance from age 20–25, every 6 m to 5 y guided by Spigelman stage. Endoscopic resection in case of FGPs with dysplastic features and in GAs ≥ 5 mm.	Gastroduodenoscopy surveillance from age 30–35 y, every 6 m to 5 y guided by Spigelman stage. Endoscopic resection in case of FGPs with dysplastic features and in GAs ≥ 5 mm.	In the case of polyp with dysplasia: total gastrectomy. Absence of dysplasia: consider prophylactic gastrectomy in patients between 30 and 35 y or 5 y before the presence of gastric cancer in the youngest family member or annual gastroscopic follow-up.
